# Infrared and Visible Image Fusion through Details Preservation

**DOI:** 10.3390/s19204556

**Published:** 2019-10-20

**Authors:** Yaochen Liu, Lili Dong, Yuanyuan Ji, Wenhai Xu

**Affiliations:** School of Information Science and Technology, Dalian Maritime University, Dalian 116026, China; yaochen_liu1114@dlmu.edu.cn (Y.L.); yuanyuanji@dlmu.edu.cn (Y.J.);

**Keywords:** image fusion, guided filter, details, CNN, DCT

## Abstract

In many actual applications, fused image is essential to contain high-quality details for achieving a comprehensive representation of the real scene. However, existing image fusion methods suffer from loss of details because of the error accumulations of sequential tasks. This paper proposes a novel fusion method to preserve details of infrared and visible images by combining new decomposition, feature extraction, and fusion scheme. For decomposition, different from the most decomposition methods by guided filter, the guidance image contains only the strong edge of the source image but no other interference information so that rich tiny details can be decomposed into the detailed part. Then, according to the different characteristics of infrared and visible detail parts, a rough convolutional neural network (CNN) and a sophisticated CNN are designed so that various features can be fully extracted. To integrate the extracted features, we also present a multi-layer features fusion strategy through discrete cosine transform (DCT), which not only highlights significant features but also enhances details. Moreover, the base parts are fused by weighting method. Finally, the fused image is obtained by adding the fused detail and base part. Different from the general image fusion methods, our method not only retains the target region of source image but also enhances background in the fused image. In addition, compared with state-of-the-art fusion methods, our proposed fusion method has many advantages, including (i) better visual quality of fused-image subjective evaluation, and (ii) better objective assessment for those images.

## 1. Introduction

Image fusion is an essential technique for information fusion, which has been widely utilized in practical application such as target detection, industrial production, military and biomedical science. Especially in industrial production, infrared and visible image fusion is a reliable tool of surveillance, so it has become an active topic in the computer vision research [1,2,3]. Visible image is consistent with human visual perceptions characteristics. However, due to the influence of complex environment, visible image often suffers from loss of contrast and scene information. Infrared image is not easily affected by the external environment, but the texture is poor. Therefore, the key problem of visible and infrared image fusion is to combine with the source images features to generate the fused image, which contains high-quality details for helping subsequent processing and decision-making.

In recent decades, many image fusion methods have been proposed by different schemes, which can be mainly divided into five categories: subspace-based methods, multi-scale transform methods, sparse representation methods, saliency-based region methods, and deep learning methods. The first category means using subspace-based method [4,5]. For example, Bouwmans et al. [4] utilize robust principal component analysis (RPCA) via decomposition into low-rank plus sparse matrices to offer a framework for image processing, which gives a inspiration to the research of image fusion. Cvejic et al. [5] convert source images to the independent component analysis (ICA) domain, and then the ICA coefficients from given regions are weighted by the Piella fusion metric. This kind of method has the advantage of computational efficiency, but the adaptability is poor. The second category means using multi-scale transform method [6,7]. Zhang et al. [6] study a generic image fusion framework based on multiscale decomposition, and then this framework is widely utilized to study image fusion methods such as discrete wavelet transform (DWT). Chai et al. [7] use quaternion wavelet transform (QWT) to decompose images into multi-scale, and then the contrast and the energy of coefficient are utilized to fuse the low frequency subbands and the high frequency subbands respectively. This kind of method conforms to human visual characteristic, but the disadvantage is that only the common features such as edge information can be retained in the fused image. The third category means using sparse representation method [8,9]. Zhang et al. [8] propose a survey on sparse representation fusion methods, which proves sparse representation strategy that is an effective tool for integrating the feature of the human visual system. Yang et al. [9] use sparse coefficients to represent the source images, then both sparse coefficients and the dictionary are utilized to reconstruct the fused image. This kind of method can extract the key features of the source images, however, these fusion methods cannot simultaneously preserve the details of infrared and visible images. The fourth category means using the saliency-based region method [10,11]. Meher et al. [10] present a review of existing region-based image fusion method. Zhou et al. [11] study a target-aware decomposition and parallel gradient fusion method to maintain the high brightness region of infrared image in the fused image. This kind of method can effectively fuse the target region, but the background reconstruction is ignored. The fifth category means using deep learning method [12,13,14]. Liu et al. [12] fully investigate the literature of image fusion method based on deep learning, and then they put forward the key problems and challenges in the future. Ma et al. [13] use a generative adversarial network to fuse infrared and visible image, which can keep both the thermal radiation in an infrared image and the textures in a visible image into the final fused image. Y. Liu et al. [14] utilize CNN to obtain a weight map and use image pyramids to fuse the infrared and visible images. This kind of method has been widely applied since CNN, which has has strong feature extraction ability, was introduced into the image fusion community, however, these methods cannot take full advantage of the extracted features.

**Figure 1:**
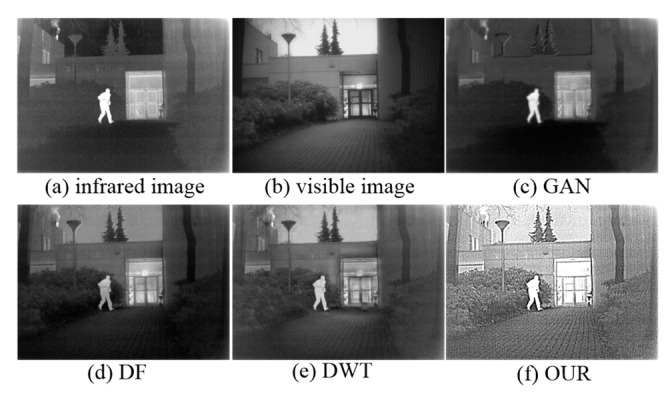
Schematic illustration of image fusion. (**a**) Infrared image. (**b**) Visible image. (**c**) Fusion result of generative adversarial network (GAN). (**d**) Fusion result of DeepFuse (DF). (**e**) Fusion result of discrete wavelet transform (DWT). (**f**) Fusion result of our proposed method.

In brief, many academics have achieved plenty of infrared and visible image fusion methods, but there are still problems to be solved. To show problems of existing fusion methods including generative adversarial network (GAN) [13], DeepFuse (DF) [15], and DWT [6], we give a representative example in Figure 1. It can been seen that the target region and rich texture are contained in Figure 1a,b, respectively. The result of GAN [13] can preserve the salient region of infrared image, but it lacks details of the source images because the extracted features can’t be effectively integrated. To a certain extent, the result of DF [15] can preserve texture, but the tiny details are totally lost in the background. In addition, texture is not uniform and the visibility is poor in the result of DWT [6]. On the contrary, our fused image not only retains the thermal radiation distribution but also enhances the details of the source images in the background.

Besides the above analysis, there are three weaknesses of existing fusion methods obstructing the obtention of high-quality details.

(1) The tiny details cannot be decomposed into the detail part. This brings about uneven texture and poor visibility in the fused image.
(2) These methods cannot extract different features of the source images, leading to the loss of various features in the fused image.
(3) The extracted features cannot be fully utilized, which cause blurring of the fused image.

Here are three proposed solutions to these problems.

(1) For the first problem, our method takes the advantage of guided filter to get the detail part, with the image containing only the strong edge of the source image as the guidance image and the source images as the input images. In this way, rich tiny details can be decomposed into the detail part.
(2) For the second problem, a rough CNN and a sophisticated CNN are designed to extract various features of the infrared and visible images respectively.
(3) For the third problem, a multi-layer features fusion strategy is proposed, which combines the advantages of DCT. In this way, the significant features can be highlighted and the details can be enhanced.

The rest of this paper is organized as follows: the proposed fusion image method is given in Section 2, including image decomposition, feature extraction and fusion rule. The comparison and analysis of the experimental results are shown in Section 3. Finally, the conclusions of this paper are stated in Section 4.

## 2. Image Fusion Method through Details Preservation

**Figure 2:**
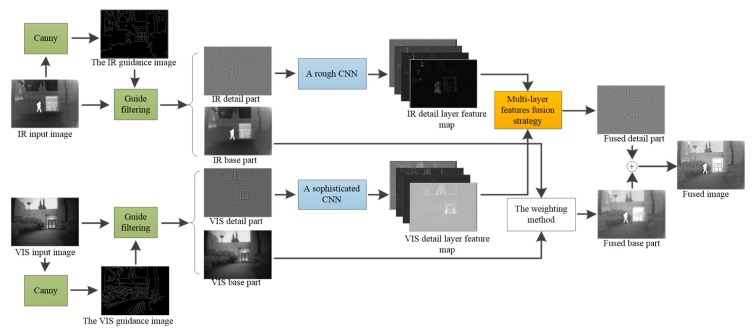
The framework of the proposed method.

In this section, a detailed description of the proposed fusion method is given. The fusion framework is presented in Figure 2. Firstly, the source images are decomposed into base parts and detail parts by guided filter, with the image that only contains strong edge information of the source images obtained by the canny operator as the guidance image and the source images, as the input images. Secondly, a rough CNN and a sophisticated CNN are designed to extract the features of infrared and visible detail parts respectively. Then a multi-layer features fusion strategy is utilized to integrate the extracted features. Moreover, the base part is fused through weighting method. Finally, the fused image is reconstructed by the adding of the fused detail and base parts.

### 2.1. Image Decomposition by Guided Filter

An image generally contains lots of different-part information, and the applied research of image is sometimes limited to the phenomenon of one part or some parts. Therefore, it is necessary to decompose the images into different parts, which not only eliminates the influence of other parts on image processing results, but also simplifies the complexity and difficulty of image processing. In this paper, the source images are decomposed into the detailed part containing details and the base part containing gray distribution by guided filter.

There are many methods to decompose image into the base and detail parts, such as gaussian filter et al. [16,17,18]. However, these methods bring ringing artifacts due to the blur of strong edge. However, this phenomenon can be avoided by edge-preserving filters such as bilateral filter [19], guide filters [20] since they can preserve strong edge. In which, guided filter is widely used in image decomposition, which has short computation time and overcomes the gradient flip of bilateral filter.

Most scholars use guided filter to decompose images by using the source images as both the guidance image and the input image, which fails to smooth the tiny details within textured region. In order to solve the above problem, different from most decomposition method by guided filter, we utilize canny operator to obtain the image containing the strong edge of the source images as the guidance image. The decomposition method can be divided into two steps: getting the guidance image and image decomposition using guided filter.

The first step: Getting the guidance image. As we all know, the characteristic of the guided filter is that the output image is similar to the input image as a whole, but the texture is similar to the guidance image. Therefore, we need to obtain the guidance image, which contains only the strong edge of the source image but no other interference information. Through this operation, the output image also keeps the strong edge and the area with rich details will becomes smoother. At present, there are many methods to extract strong edge, such as roberts operator, prewitt operator and canny operator. Roberts operator is more sensitive to noise. Prewitt operator is easy to lose edges with smaller amplitude. However, canny operator is a multi-order operator with filtering, enhancement and detection functions, which is better than other edge detection method [21]. We use the canny operator to determine the strong edge pixels of the image, and then set the gray levels of other pixel positions to 0, thus obtaining the guidance image, as shown in (1):

(1) $$W_{n}=Canny(I_{n})$$

where Canny( ) is the canny operator, $I_{n}$ is the nth source image, W is the guidance image and n={1,2}.

The second step: Image decomposition using guided filter. Use $W_{n}$ as the guidance image, $I_{n}$ as the input image, as shown in (2):

(2) $$P_{n}=Guider_{r_{n},\varepsilon_{n}}(I_{n},W_{n})$$

where $P_{n}$ is the base parts, Guider( ) is guided filter, $r_{n}$ and $\varepsilon_{n}$ are the parameters of guided filter.

After the output image is obtained, the detail part can be obtained by subtracting the output image from the source image, as shown in (3):

(3) $$D_{n}=I_{n}-P_{n}$$

where $D_{n}$ is detail part.

**Figure 3:**
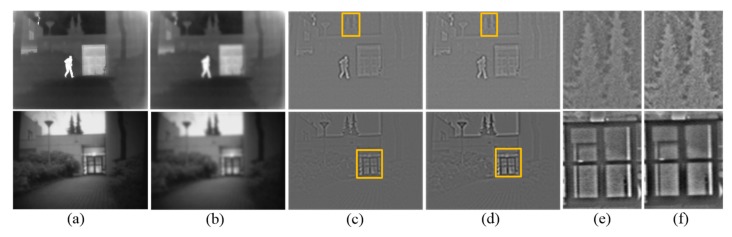
Comparison of different decomposition methods. (**a**,**c**) are the base part and the detail part obtained by common method. (**b**,**d**) are the base part and the detail part obtained by our proposed method. (**e**) is the local amplification effect of the yellow box of the (**c**). (**f**) is the local amplification effect of the yellow box of the (**d**). From top to bottom the figures are respectively infrared image and visible image.

To show intuitive results on the decomposition performance, we give an example in Figure 3. It can been seen that the textured region is not smooth enough in Figure 3a. On the contrary, the strong edges are preserved and the texture region is very smooth in Figure 3b. In addition, the details look blurred in Figure 3c, but Figure 3d obtains rich tiny details. The yellow box of Figure 3c,d are zoom out for better analyses, which are given in Figure 3e,f respectively. We can see that the treetop and door frame area in Figure 3f are clearer than Figure 3e. Through the above analysis, it is shown that the decomposition method in this paper can extract richer texture details. Experiments show that better results can be achieved by choosing fixed parameters $r_{1}=r_{2}=3$, $\varepsilon_{1}=0.1$ and $\varepsilon_{2}\;=\;0.61$.

### 2.2. Fusion of Detail Parts Based on CNN and DCT

**Figure 4:**
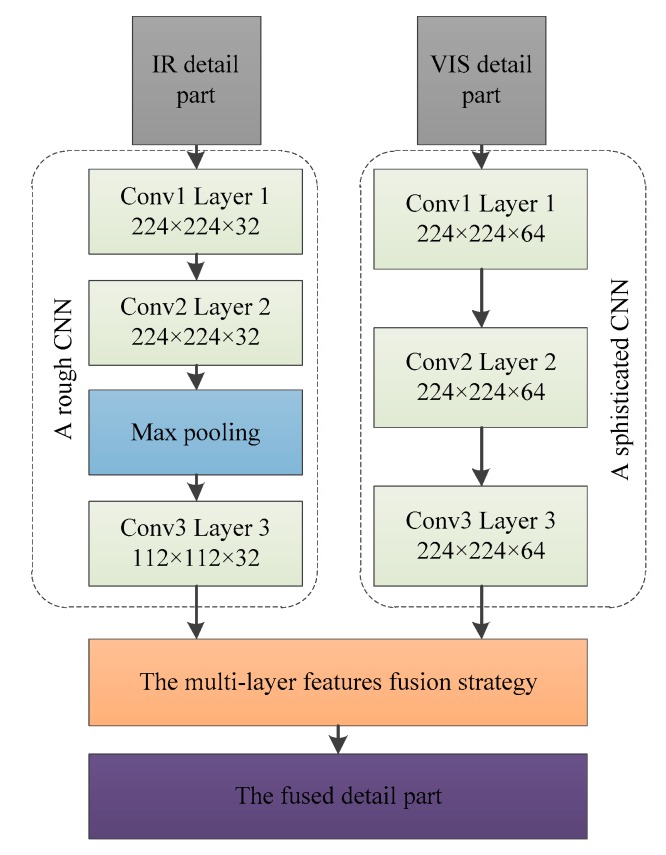
The procedure of the detail part fusion.

According to our proposed decomposition method, we can know that the infrared detail part contains noise and fuzzy texture, but the edges of the salient region are clear. In addition, we also investigate that the visible detail part has abundant tiny details that conform to human vision. Figure 4 shows the whole detail part fusion scheme, where the input infrared detail part and visible detail part are generated from our proposed decomposition method. In order to effectively fuse image detail layer, we design a rough CNN and a sophisticated CNN to extract the features of the detail parts. Then, we design a multi-layer features fusion strategy for integrating features.

#### 2.2.1. Infrared Detail Layer Features Extraction by Rough CNN

Since infrared sensors are insensitive to scene details, the details are poor in the infrared detail layer. Specifically, the infrared detail layer can contain the contour information of the salient region, while the background includes lots of noise. According to the characteristics of infrared detail parts, a rough CNN is designed to extract multi-layer features, which consists of three convolutional layers and two max-pooling layers. For the number of convolution layers, VGG network [22] has proved that deeper network structure is more helpful for comprehensive feature extraction. However, with the increase of network depth, it will lead to the waste of computing resources. Therefore, it is very important to choose the appropriate number of convolution layers. In [14,23], three convolution layers are presented for feature extraction, so we contain three convolution layers in network. For pooling layer selection, the first pooling layer is placed in the middle of the three convolution layers, which has the functions of reducing computational complexity, retaining salient edges and ignoring noise in the background. The second pool layer is placed behind three convolution layers, which has the function of extracting main features to prepare for the full-connected layer (not shown in Figure 4). For the size of convolution layers, using high-resolution images as input data can improve network performance. However, high-resolution images will increase the computational cost of the model and lead to the prolongation of the overall training time of the network. Through the above analysis and experimental tests, the first convolution layer is set to 224 × 224 × 32. Furthermore, because the infrared detail part contains lots of noise and blurred texture in background, we don’t deepen the depth of the convolutional layer to compensate for the missing tiny features after the pooling layer. The configuration of network mentioned above is summarized as shown in Figure 4. In addition, it should be noted that the kernel size and stride of each convolutional layer are set to 3 × 3 × 1.

#### 2.2.2. Visible Detail Layer Features Extraction by Sophisticated CNN

The visible sensor can acquire clear image details, which is more suitable for human visual observation, so the visible detail layer contains rich and useful features. According to the characteristics of visible detail layers, a sophisticated CNN is designed to extract multi-layer features. Specifically, compared with the infrared detail layer CNN, we abandon the first pooling layer and only retain the second pool layer because the first pooling layer loses some minute features. In addition, in order to extract rich features, the convolution layer is set to 224 × 224 × 64.

#### 2.2.3. Training

In the training phase, we mainly consider that each convolution layer can extract the rich features. Selecting different training data for different purposes can effectively train the model. Therefore, different from other deep learning-based fusion methods [14,24], we propose that infrared and visible images are used as the training data of the infrared and visible detail parts networks, respectively. The 105 pairs of infrared and visible images from TNO database are selected as training data. However, it is insufficient to train a good mode, so we rotate the images 90°, each image is then randomly divided into 50 frames with a size of 224 × 224. After this operation, we can obtain 22,500 pairs of training data to expand the dataset. The task of image classification based on CNN has been proved to be able to extract image features, and has been applied to the field of image fusion [14,23], so we use the same method to train models. Since the training data are mainly divided into two categories, including people and excluding people, the infrared images containing people are set to the same label, while the other infrared images are set to the same label. For visible images, the same approach is adopted. In addition, we set the learning rate to10^−4^ and train the network for 50 epochs. The loss and optimization function have an important impact on the training efficiency and effectiveness of the model. The cross-entropy loss is used as the loss function to calculate the model error, and adaptive moment estimation (Adam) is used as the optimization function to optimize the parameters.

#### 2.2.4. Design of Multi-Layer Feature Fusion Strategy by DCT

In an existing method based on deep learning [24,25], only the last layer or largest layer is used, which loses many useful features in the fused image. We propose a multi-layer features fusion strategy to overcome this problem. The multi-layer feature fusion strategy contains three steps: obtaining feature saliency maps, reconstructing new detail parts and merging new detail parts.

The first step: obtaining feature saliency maps. Each convolution layer is charged with specific responsibilities [26], so we add feature maps contained in each convolution layer, and then normalize them to generate feature saliency maps. The inspiration of this operation comes from itti’s visual attention model [27]. Normalized operation can compare the maximum activity to the average activity. When the difference is large, the most active position will be highlighted. When the difference is small, the feature map contains nothing unique, as shown in (4) and (5):

(4) $$A_{1}^{i}=Nor(\sum_{m=1}^{32}\Phi_{1}^{m,i})$$

(5) $$A_{2}^{i}=Nor(\sum_{l=1}^{64}\Phi_{2}^{i,l})$$

where $A_{1}^{i}$ and $A_{2}^{i}$ are the ith feature saliency map of infrared and visible detail parts. Nor(⋅) is a normalized operation, $\Phi_{1}^{m,i}$ is the feature maps of the infrared detail part extracted by ith layer and m is the channel number of the ith layer. $\Phi_{2}^{i,l}$ is the feature maps of the visible detail part extracted by ith layer and l is the channel number of the ith layer. 32 and 64 are the number of infrared and visible feature maps, respectively.

**Figure 5:**
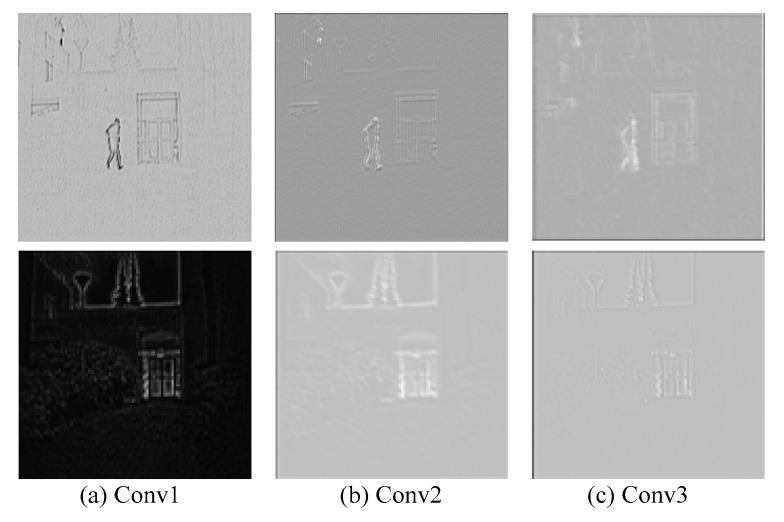
Feature saliency map of each convolutional layer. “conv1”, “conv2” and “conv3” denote the first, second and third convolutional layer, respectively. From top to bottom, the figures are infrared and visible feature saliency maps, respectively.

Figure 5 shows feature saliency maps of each convolutional layer. Each feature saliency map is normalized to the range of [0, 1]. As shown in Figure 5a,b, it can be seen that the significant details in the infrared detail layer can be extracted, and the weak features in the visible detail layer can be accurately captured such as grass. Figure 5c shows that the infrared feature saliency maps retain the features of salient edges, but the weak features are ignored due to the influence of the pooling layer. However, the visible feature saliency maps focus on active details. It can be demonstrated that the infrared feature saliency maps not only preserve the salient features but also ignore the noise feature. Meanwhile, the visible feature saliency maps can obtain the features of tiny details.

The second step: reconstructing new detail parts. Since feature saliency maps are different from the source image in size, the feature saliency maps are interpolated into the size of the source images. Then weight maps of infrared and visible detail parts are obtained, as shown in (6):

(6) $$S_{n}^{i}(x,y)=\frac{\overset{\wedge }{A_{n}^{i}}(x,y)}{\sum_{n=1}^{2}\overset{\wedge }{A_{n}^{i}}(x,y)}$$

where $S_{n}^{i}(x,y)$ is in the range of [0,1]. $\overset{\wedge }{A_{n}^{i}}$ is feature saliency maps after interpolation.

Then, four new detail parts $d_{n}^{i}(x,y)$ are reconstructed, as shown in (7):

(7) $$d_{n}^{i}(x,y)=S_{n}^{i}(x,y)\times D_{n}(x,y)$$

The third step: merging new detail parts. Multi-scale transformation is used to merge detail parts because the fusion method based on multi-scale transformation is proved to be consistent with human vision such as contourlet transform (CT) [28], DCT [29], nonsubsampled contourlet (NSCT) [30]. CT loses image information after sampled, so NSCT is widely used in image processing. However, NSCT takes a lot of computation. In the multi-scale transformation method, the advantages of DCT are that the image information is not lost and the computational complexity is not as much as NSCT. In addition, we can easily achieve the purpose of denoising by DCT because it has excellent energy compactness properties. Therefore, we use DCT to merge new detail parts, as shown in (8):

(8) $$D(x,y)=\sum_{i=1}^{4}\alpha_{i}\times DCT(d_{1}^{i}(x,y)+d_{2}^{i}(x,y))$$

where DCT(⋅) is the DCT operation, $\alpha_{i}$ is the fusion weight, and D(x,y) is the fused detail. In this paper, the common data set TNO is used as an experimental sample for fusion experiments, and it is found that when the parameter $\alpha_{i}$ in [1, 2] is obtained, a better fusion effect can be obtained.

**Figure 6:**
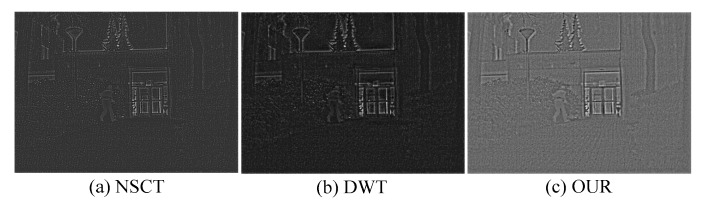
Comparison of the three detail part fusion methods. (**a**) the result of nonsubsampled contourlet (NSCT), (**b**) the result of DWT and (**c**) the result of our method.

As shown in Figure 6, compared the three detail part fusion methods. Figure 6a,b are unable to preserve the feature of details so that the image looks blurry. On the contrary, Figure 6c emphasizes the features of the source images such as the tiny floor texture and the obvious edge of human because our feature extraction method can obtain rich feature information. In addition, Figure 6c is also very natural because the multi-layer feature fusion strategy in this paper can effectively utilize the extracted features. Through the above analysis, it is shown that the detail parts fusion rule in this paper can preserve the details of source images to the fused detail part.

### 2.3. Weighting Method Fusion Base Parts

For the base part fusion, Ma et al. [31] use the visual attention model to obtain the fused base part, Zhou et al. [11] utilize L_0 filter for decomposing target region as the fused base part. However, these methods are inefficient and hard to implement. The base part mainly contains the gray distribution and redundant information of source images. Therefore, we can fuse the base part well by weighting method [32], which is easy to implement and fast in operation, as shown in (9):

(9) $$P(x,y)=\sum_{n=1}^{2}\beta_{n}P_{n}(x,y)$$

where P(x,y) is the fused base part, and $\beta_{n}$ are the weight coefficient. When added to $\beta_{n}$, the gray level of fusion base part P(x,y) will be increased, but may saturate the gray levels because its excessive overshooting. When reduced to $\beta_{n}$, the fusion base part contrast can be decreased due to the reduction of gray levels. Experiments show that better results can be achieved by choosing fixed parameters $\beta_{1}\;=\;0.8$, $\beta_{2}\;=\;0.6$.

### 2.4. Two-Scale Image Reconstruction

The reconstruction steps are as follows:

Step 1: according to Section 2.2, it shows that this paper uses CNN to extract the features of detail parts, and uses the advantages of DCT to design a multi-layer features fusion strategy to get the fused detail part D(x,y).
Step 2: according to Section 2.3, it shows that P(x,y) is the fused base part by weighting method.
Step 3: we get the fused image F(x,y), as shown in (10):
  (10) F(x,y)=P(x,y)+D(x,y)

According to Equation (3), we know that the detail part was obtained by subtracting the base part from the source images, so reconstruction image can be obtained through adding the fused base part P(x,y) and the detail part D(x,y).

## 3. Experimental Results

In this section, for effectively evaluating the performance of our proposed fusion method, public data is used as test images. In addition, we compared with state-of-the-art methods from both subjective and objective performance respectively, and the computational costs are also discussed. The experimental settings and the results of all fusion methods are detailed as follows.

### 3.1. Experimental Settings

#### 3.1.1. Image Sets

**Figure 7:**
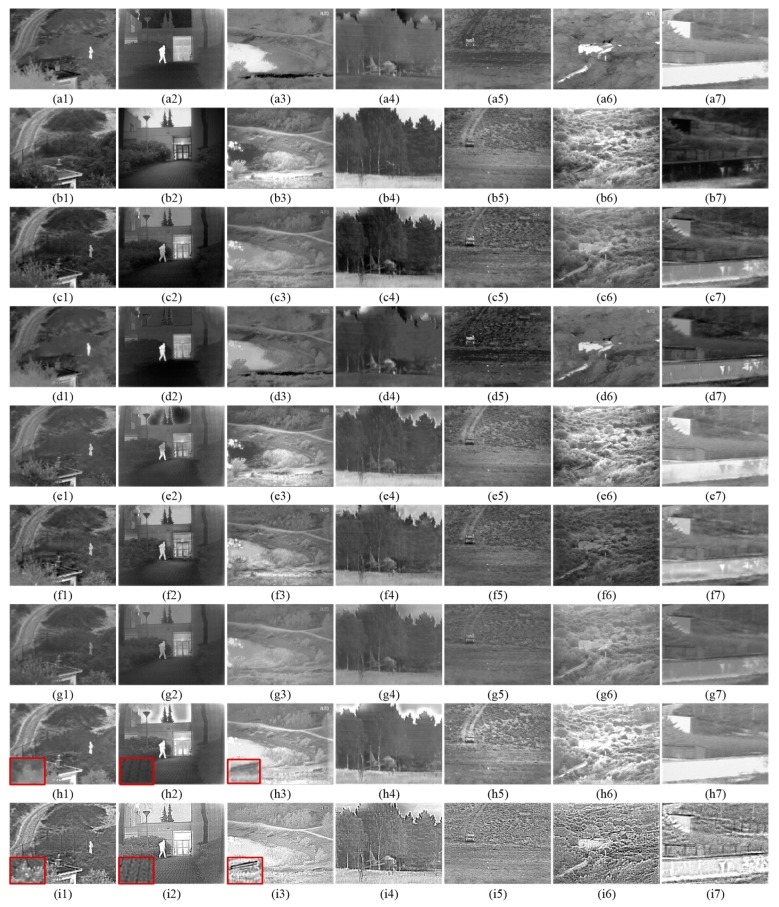
Subjective performance results on seven typical infrared and visible image pairs from the TNO database. From left to right: “Camp”, “Kaptein”, “Lake”, “Airplane”, “Fennek”, “Bunker” and “Wall”. From (**a1**–**i7**): infrared image, visible image, results of DF, GAN, GFF, DWT, DCT, QBI, and OUR.

In experimenting, we selected seven pairs of visible and infrared images as the experimental sample, which were collected from TNO datasets [12]. Each image pair is pre-registered and has different scenes. The first two rows of Figure 7 show the seven pairs of images arranged from left to right including “Camp”, “Kaptein”, “Lake”, “Airplane”, “Fennek”, “Bunker” and “Wall”. The image size is 360 × 270, 620 × 450, 768 × 576, 595 × 328, 749 × 551, 749 × 576 and 280 × 280, respectively.

#### 3.1.2. Compared Methods

To show the advantage of the proposed fusion method, six representative fusion methods are chosen for comparison including GAN [13], DF [15], guided filtering-based fusion (GFF) [33], quadtree-bezier interpolation (QBI) [34], DWT [6] and DCT [29]. Among these compared methods, GAN [13] and DF [15] utilizes convolution neural network to extract the source images and could achieve good results. GFF [33] uses guided filter to obtain weight maps for visible and infrared images fusion. QBI [34] firstly utilizes quadtree decomposition and Bezier interpolation to extract infrared features, and then the visible image and infrared features are integrated to obtain the result. DCT [29] and DWT [6] are the multi-scale fusion method, which are widely used in the field of image fusion. In addition, the publicly available codes were used to implement all six comparative methods, and the parameter size is the default value of the source code.

#### 3.1.3. Computation Platform

All fusion methods are implemented on a PC-Windows 10 platform with Inter (R) Core (TM) i7-8700K @ 3.70 GHz processor, 16 GB RAM, and CeForce GTX 1080 Ti. Among them, our proposed method, DF, and GAN are performed on graphics processing unit (GPU), while all the other methods are performed on central processing unit (CPU).

### 3.2. Subjective Performance Evaluation

The subjective performance is based on human visual system to evaluate the quality of fusion image, which has good reliability. In order to verify the subjective performance of our proposed fusion method in different scenarios, Figure 7 shows the results of all fusion methods. In which, the first two rows are infrared and visible image respectively, and the last row corresponds to the fused images of our proposed fusion method and the results of all six comparative methods are shown in the rest six rows.

From Figure 7, we can see that all infrared and visible image fusion methods have accomplished the task of fusing source images information to a certain degree. However, experiments prove that our fused images are clearer than the other six fusion results. Furthermore, the results of our fusion method are neither over-enhanced nor under-enhanced. Our image decomposition operation plays a key role in our fusion method, which affects the extraction of details from the source images. If abundant tiny details are not decomposed into detail parts, the texture will be blurred in fused image. In the results of DWT, although salient information of infrared image is preserved, details become fuzzier because the tiny details are not decomposed into detail parts. For example, the grass in Figure 7f1 and the floor in Figure 7f2 are too dark. In addition, the subjective comparison demonstrates that our fused images conform to human visual senses because the features of the source images were sufficiently extracted by our method. We can see that DF method’s results cannot preserve the feature of infrared image, so the contrast of fused images is very low. Then GFF method’s result abandons the source image regional feature, such as the sky distortion in Figure 7e2,e4. Fortunately, our fused image is very natural at the same place because robustness of feature extraction is used. Meanwhile, our multi-layer feature fusion strategy can effectively unveil details and preserve natural appearance in our fused image. Although GAN method is able to preserve the gray distribution from infrared image, its results are not as nature as our method because GAN ignores the exploitation of the feature information in the visible image. Additionally, because various features were extracted, our method can highlight the saliency region in the fused images and the background regions are clearer than the result of DCT. QBI method is better than other five comparative methods in detail preservation. But our fusion results have better resolution and clarity in region of texture. For example, in Figure 7h1, the leaf in a red box has a clearer outline in our result. In Figure 7h2, the salient region is highlighted in two fused images, but artifacts appear in the sky and the floor in the red box is unclear in the result of QBI. In Figure 7h3, the chair in the red box has a better contrast than QBI. Of course, you can also observe similar regions in the other five comparative methods. All these phenomena state that our proposed method has better performance in subjective vision.

### 3.3. Objective Performance Evaluation

Objective performance evaluation depends on the evaluation metrics that are given by the mathematical model, which is not disturbed from human visual characteristic and mental state. Therefore, besides subjective performance evaluation, objective metrics are adopted to measure the objective performance. The objective performance is an essential means that can evaluate the characteristic of fused images, but a single metric cannot fully reflect the image characteristics. In order to evaluate image features effectively, we will employ six typical objective metrics for subjective performance, including visual information fidelity (*VIF*) [35], entropy (*EN*) [36], mean gradient (*MG*) [37], mutual information (*MI*) [38], spatial frequency (*SF*) [39] and standard deviation (*SD*) [40]. The six metrics are defined as follows.

(1) Entropy

*EN* reflects the richness of the image information. A large *EN* metric means that the fused image contains rich information. Therefore, *EN* is usually used to evaluate the performance of fusion method. *EN* is defined as follows:

(11) $$EN=-\sum_{l=0}^{L-1}p_{i}\log p_{i}$$

where *L* is gray levels, $p_{i}$ is the normalized histogram of the corresponding gray level in the fused image.

(2) Visual information fidelity

*VIF* is used to measure image visual information fidelity, which depends on natural scene statistical model, image signal distortion channel and human visual distortion model. A large *VIF* metric indicates that the fused image is very consistent with human visual senses.

(3) Mean gradient

*MG* reflects the ability of image to express texture variation. A large *MG* metric indicates that the fused image has abundant edge and texture information. For an input image F(i,j), *MG* is defined as follows:

(12) $$MG=\frac{1}{\left(m-1)(n-1\right)}\sum_{i=1}^{m}\sum_{j=1}^{n}\sqrt{\frac{\left({\left(F(i,j)-F(i-1,j)\right)}^{2}+{\left(F(i,j)-F(i,j-1)\right)}^{2}\right)}{2}}$$

where m and n are image sizes.

(4) Mutual information

*MI* shows the amount of information that is transferred from source image to the fused image. A large *MI* metric means that rich information is transferred from infrared and visible to the fused image. *MI* is defined as follows:

(13) $$MI=MI_{A,F}+MI_{V,F}$$

(14) $$MI_{X,F}=\sum_{x,f}p_{X,F}(x,f)\log\frac{p_{X,F}(x,f)}{p_{X}(x)p_{F}(f)}$$

where $MI_{A,F}$ and $MI_{V,F}$ are the amount of information that is transferred from infrared and visible to the fused image, respectively. $p_{X,F}(x,f)$ is the joint histogram of the source image X and the fused image F. $p_{X}(x)$ and $p_{F}(f)$ are the marginal histograms of X and F, respectively.

(5) Spatial frequency

*SF* reflects the sharpness and clarity of the fused image. A large *SF* metric illustrates that the fused image has good definition. *SF* is defined as follows:

(15) $$SF=\sqrt{RF^{2}+CF^{2}}$$

(16) $$RF=\sqrt{\sum_{i=1}^{M}\sum_{j=1}^{N}{\left(F(i,j)-F(i,j-1)\right)}^{2}}$$

(17) $$CF=\sqrt{\sum_{i=1}^{M}\sum_{j=1}^{N}{\left(F(i,j)-F(i-1,j)\right)}^{2}}$$

(6) Standard deviation

*SD* measures the distribution structure of gray level. A large *SD* metric shows that the fused image has wide gray distribution, *SD* is defined as follows:

(18) $$SD=\sqrt{\sum_{i=1}^{M}\sum_{j=1}^{N}{\left(F(i,j)-\mu \right)}^{2}}$$

where μ is the gray average.

The objective performance of the compared methods and the proposed method are listed in Table 1. It can been seen that the proposed methods have a higher average evaluation value than the compared methods. Specifically, because our method can extract the rich features of source images details very well, *MG* and *SF* are always the best comparative methods. In addition, we can see that *MI* and *EN* also achieve high performance, except that GFF respectively achieves the highest value of metrics *MI* and *EN* on the “Laker” and “Bunker” source images because GFF loses the information of infrared image in the fused image. On the premise of combining visible and infrared image information, the performance of *EN* shows that our method contains more information than other fusion methods. The performance of *MI* shows that the information of the source image is adequately transmitted to the fusion image. In the result of *SD*, our fusion method still has a good performance, because the decomposition method in this paper decomposes rich tiny details into the detail parts so that the fusion image has good gray distribution. Finally, due to the influence of noise, the *VIF* of our method is not the best, but our fused images still have great visual effect and keep excellent correlation with the source images.

### 3.4. Computational Costs

We must measure the computational cost of fusion methods, except the subjective and objective performance evaluation, which determines the actual application value of the method. The running time is used to evaluate the computational cost of all fusion methods. The infrared and visible image pairs in Figure 7 are taken as an example for computational costs analysis. As shown in Table 2, the results of QBI and DCT cost less running time compared to other methods because they are not complex and have high computational efficiency. GFF wastes a lot of times on the acquisition of weight maps. In DWT, multiscale transform is complex, costing more time. In addition, comparing other deep learning methods, GAN spends less time because the size of convolution layer is small. The running time of DF is wasted in solving deep feature, so it takes the longest time in all methods. Our proposed method is not the fastest because the size of convolution layers is larger. However, considering the high-quality fusion image, our method is still an effective fusion method.

## 4. Conclusions

In this paper, we presented a novel infrared and visible image fusion method through details preservation, which can obtain excellent details information and simultaneously retain the gray distribution information of the source images. Experiments on TNO datasets indicate that our fused images look like sharpened images with abundant details, which is beneficial for observing the actual scene. The subjective and objective performance evaluation reveals that our fusion method can obtain great visual effects, and preserve a large amount of information in the source image. In the future, an adaptive fusion framework will be built and the stability of the method will be enhanced.

## Figures and Tables

**Table 1:** Objective metrics of seven methods. For all six metrics, larger values indicate better performance.

Source Image	Index	DF	GAN	GFF	QBI	DWT	DCT	OUR
Camp	EN	6.8369	6.5921	6.3337	6.7693	6.7044	6.3127	7.0462
	MG	5.6419	3.1139	4.2137	5.0931	5.0542	3.3768	11.7701
	MI	13.6737	13.1841	12.6675	13.5386	13.4087	12.6255	14.0923
	SD	34.4917	25.8433	25.3713	33.7623	27.5241	24.6854	36.8892
	SF	11.1869	6.3977	9.0002	10.8049	9.9757	6.7636	22.9205
	VIF	0.5707	0.3215	0.5882	0.6896	0.5539	0.5873	0.6758
Keptein	EN	6.9733	6.7229	6.8564	7.1692	6.9279	6.5259	7.3641
	MG	6.0872	1.9692	3.5963	4.7491	4.3282	2.8373	18.1836
	MI	13.9466	13.4458	13.7128	14.3384	13.8557	13.0519	14.4281
	SD	43.6383	34.9727	33.2555	49.5339	41.3485	31.5369	54.9976
	SF	12.3499	4.3892	7.0715	9.2726	8.0446	5.2636	32.0286
	VIF	0.5716	0.3975	0.7058	0.9552	0.7783	0.7079	1.0037
Laker	EN	6.8733	6.5194	7.3661	7.0391	6.9733	6.5385	6.9747
	MG	6.0659	2.8229	4.6354	4.8585	4.4619	2.7834	19.1483
	MI	13.7466	13.0388	14.7323	14.0781	13.9525	13.0771	14.5495
	SD	30.4503	30.2064	43.9306	40.484	35.5537	24.1975	44.0385
	SF	12.2313	6.2371	11.4587	10.4112	9.9456	6.2536	35.7208
	VIF	0.4825	0.5303	0.8905	0.8532	0.7118	0.6406	0.8816
Airplane	EN	7.0575	6.0773	6.4477	6.7303	6.6552	6.1811	7.2813
	MG	4.6496	1.4983	2.2827	2.7531	2.4319	1.5607	9.4955
	MI	14.1151	12.1546	12.8955	13.4606	13.3103	12.3621	14.5661
	SD	55.9542	20.9171	44.6439	47.9522	41.3763	30.4096	46.9162
	SF	11.0442	3.3018	5.2581	7.1919	5.4297	3.3898	20.1914
	VIF	0.8279	0.5059	0.7515	0.8799	0.7828	0.6889	1.1583
Fennek	EN	6.2403	6.1625	6.3859	6.6034	5.9661	5.3997	7.3157
	MG	9.4777	4.1883	4.3086	8.4251	4.9379	3.2229	26.3613
	MI	12.4807	12.3251	12.7719	13.2068	11.9321	10.7994	14.1314
	SD	19.1077	18.6671	20.4052	25.2119	15.4756	10.4274	39.9587
	SF	16.2758	7.1355	7.4061	14.3185	8.3821	5.4628	43.5421
	VIF	0.4431	0.4852	0.8333	1.1037	0.5843	0.5822	0.7964
Bunker	EN	7.0003	6.4505	7.4894	6.9885	6.3587	6.7014	7.1975
	MG	8.7679	3.7572	7.0454	8.2489	4.2099	4.1189	23.0965
	MI	14.0061	12.9011	14.9787	13.9771	12.7175	13.4028	14.5949
	SD	31.6247	26.4067	45.271	39.6577	20.4897	25.5854	47.9166
	SF	15.5211	6.9616	13.3655	15.1211	7.6618	7.5333	40.4068
	VIF	0.3636	0.3651	0.8586	0.7384	0.5103	0.5241	0.6131
Wall	EN	7.1508	6.7714	6.9342	6.6606	6.8639	6.3696	7.2837
	MG	4.9838	3.9107	4.0493	4.4726	4.2235	2.7378	16.4612
	MI	14.3016	13.5428	13.8684	13.3211	13.7277	12.7393	14.5674
	SD	41.5795	36.4328	41.3634	45.7268	38.3868	24.0783	46.3583
	SF	9.3014	7.2743	8.0142	8.7172	7.7863	5.0341	29.1331
	VIF	0.7201	0.2913	0.7068	0.8944	0.6593	0.6471	0.8073

**Table 2:** Running time comparison of seven methods on the “Camp”, “Kaptein”, “Lake”, “Airplane”, “Fennek”, “Bunker” and “Wall”. Our method, DF, and GAN are performed on GPU, while all the other methods are performed on CPU. Each value denotes the mean of running times of a certain method on a dataset (unit: second).

Method	Camp	Kaptein	Lake	Airplane	Fennk	Bunker	Bunker
Dense	2.074	2.171	2.189	2.196	2.122	2.139	2.191
Gan	0.154	0.171	0.253	0.184	0.102	0.137	0.146
GFF	0.556	0.486	0.583	0.421	0.305	0.587	0.561
QBI	0.038	0.051	0.082	0.081	0.041	0.062	0.051
DWT	0.581	0.537	0.638	0.473	0.412	0.638	0.605
DCT	0.013	0.056	0.090	0.059	0.007	0.095	0.153
OUR	1.914	1.963	2.121	1.831	1.675	1.598	2.098
